# Effectiveness of Routine BCG Vaccination on Buruli Ulcer Disease: A Case-Control Study in the Democratic Republic of Congo, Ghana and Togo

**DOI:** 10.1371/journal.pntd.0003457

**Published:** 2015-01-08

**Authors:** Richard Odame Phillips, Delphin Mavinga Phanzu, Marcus Beissner, Kossi Badziklou, Elysée Kalundieko Luzolo, Fred Stephen Sarfo, Wemboo Afiwa Halatoko, Yaw Amoako, Michael Frimpong, Abass Mohammed Kabiru, Ebekalisai Piten, Issaka Maman, Bawimodom Bidjada, Adjaho Koba, Koffi Somenou Awoussi, Basile Kobara, Jörg Nitschke, Franz Xaver Wiedemann, Abiba Banla Kere, Ohene Adjei, Thomas Löscher, Bernhard Fleischer, Gisela Bretzel, Karl-Heinz Herbinger

**Affiliations:** 1 Department of Medicine, School of Medical Sciences, Kwame Nkrumah University of Science and Technology (KNUST), Kumasi, Ghana; 2 Institut Médical Evangélique (IME) de Kimpese, Projet Ulcère de Buruli, Kimpese, Democratic Republic of the Congo; 3 Department of Infectious Diseases and Tropical Medicine (DITM), University Hospital, Ludwig-Maximilians-University, Munich, Germany; 4 Institut National d'Hygiène (INH), Ministry of Health, Lomé, Togo; 5 Kumasi Centre for Collaborative Research in Tropical Medicine (KCCR), Kwame Nkrumah University of Science and Technology (KNUST), Kumasi, Ghana; 6 Agogo Presbyterian Hospital, Agogo, Ghana; 7 Centre Hospitalier Régional Maritime (CHR Maritime), Tsévié, Togo; 8 Programme National de Lutte contre l'Ulcère de Buruli - Lèpre et Pian (PNLUB-LP), Ministry of Health, Lomé, Togo; 9 German Leprosy and Tuberculosis Relief Association, Togo office (DAHWT), Lomé, Togo; 10 Bernhard Nocht Institute for Tropical Medicine (BNITM), Hamburg, Germany; Fondation Raoul Follereau, France

## Abstract

**Background:**

The only available vaccine that could be potentially beneficial against mycobacterial diseases contains live attenuated bovine tuberculosis bacillus (*Mycobacterium bovis*) also called Bacillus Calmette-Guérin (BCG). Even though the BCG vaccine is still widely used, results on its effectiveness in preventing mycobacterial diseases are partially contradictory, especially regarding Buruli Ulcer Disease (BUD). The aim of this case-control study is to evaluate the possible protective effect of BCG vaccination on BUD.

**Methodology:**

The present study was performed in three different countries and sites where BUD is endemic: in the Democratic Republic of the Congo, Ghana, and Togo from 2010 through 2013. The large study population was comprised of 401 cases with laboratory confirmed BUD and 826 controls, mostly family members or neighbors.

**Principal Findings:**

After stratification by the three countries, two sexes and four age groups, no significant correlation was found between the presence of BCG scar and BUD status of individuals. Multivariate analysis has shown that the independent variables country (*p* = 0.31), sex (*p* = 0.24), age (*p* = 0.96), and presence of a BCG scar (*p* = 0.07) did not significantly influence the development of BUD category I or category II/III. Furthermore, the status of BCG vaccination was also not significantly related to duration of BUD or time to healing of lesions.

**Conclusions:**

In our study, we did not observe significant evidence of a protective effect of routine BCG vaccination on the risk of developing either BUD or severe forms of BUD. Since accurate data on BCG strains used in these three countries were not available, no final conclusion can be drawn on the effectiveness of BCG strain in protecting against BUD. As has been suggested for tuberculosis and leprosy, well-designed prospective studies on different existing BCG vaccine strains are needed also for BUD.

## Author Summary

After tuberculosis and leprosy, Buruli Ulcer Disease (BUD) is the third most common human mycobacterial disease. The only available vaccine that could be potentially beneficial against these diseases is BCG. Even though BCG vaccine is widely used, the results on its effectiveness are partially contradictory, probably since different BCG strains are used. The aim of this study was to evaluate the possible protective effect of BCG vaccines on BUD. The present study was performed in three different countries and sites where BUD is endemic: in the Democratic Republic of the Congo, Ghana, and Togo from 2010 through 2013. The large study population was comprised of 401 cases with laboratory confirmed BUD and 826 controls, mostly family members or neighbors. Considering the three countries, sex, and age, the analysis confirmed that the BCG vaccination did not significantly decrease the risk for developing BUD or for developing severe forms of BUD. Furthermore, the status of BCG vaccination was also not significantly related to duration of BUD or to time to healing of lesions. In our study, we could not find any evidence of a protective effect of routine BCG vaccination on BUD.

## Introduction

Buruli Ulcer Disease (BUD), caused by *Mycobacterium ulcerans*, is an infectious disease affecting skin, subcutanous adipose tissue, and in rare cases, bones. It is one of the 17 neglected tropical diseases as defined by the World Health Organization (WHO). BUD has been reported in 33 countries, with a major endemic focus in West and Central Africa. The exact mode of transmission of *M. ulcerans* is still unknown. However, recent studies suggest that the pathogen is acquired from the environment with different modes of transmission in different geographic areas and epidemiological settings, as shown in a systematic review [1]. Consequently, except for early case detection, confirmation, and treatment, primary measures to prevent BUD are currently lacking. Furthermore, no effective vaccine against BUD is available so far [2].

After tuberculosis and leprosy, BUD is the third most common mycobacterial disease among immunocompetent human hosts. The only available vaccine against these diseases contains live attenuated bovine tuberculosis bacillus (*M. bovis*), also called Bacillus Calmette-Guérin (BCG), named after its inventors [3]. Calmette and Guérin began their research for an antituberculosis vaccine at the Pasteur Institute in Lille, France, in 1900. The first use in humans dates from 1921, when Turpin and Weill-Hallé vaccinated infants at the Charité Hospital in Paris by oral and later also by subcutaneous and intracutaneous routes [4], [5]. From 1924 to 1928, 114,000 infants were vaccinated without serious complications, however with limited effectiveness on preventing tuberculosis [6].

From the late 1940s onward, many studies appeared providing evidence for the effectiveness of BCG for tuberculosis, with widely varying results ranging from 0% to 80% effectiveness for vaccinated adults [5], [7]. Due to these disparate results, two principal hypotheses were discussed. The first one stated that exposure to various environmental mycobacteria could itself provide some protection against tuberculosis and affect the immune system in various ways, implying that BCG could not improve greatly upon that background [5], [8]. The second hypothesis attributed the differences to variation between strains of BCG [5], [9]. It was recognized that strains produced by diverse manufacturers differed in microbiological properties, as shown in a review [10]. Hence it was not unreasonable to suggest that these might be reflected in differences in immunogenicity [5], [11]. However, in children, the effectiveness of BCG was estimated to be 50%, or even up to 80% effective in preventing tuberculous meningitis and miliary tuberculosis as shown in a meta-analysis [12] and two other publications [13]–[14].

Worldwide, over 90% of children are immunized with BCG, making it the most commonly administered vaccine, with more than 12 million doses being used each year [15]. Although BCG has been administerd to more people than any other vaccine, its history has been clouded by variable efficacy and reports of strain variability [16]. BCG has never been cloned, and there are now several different BCG seed strains in use, produced by more than 40 manufacturers [17]. Nineteen major vaccine strains are described in the literature, whereas the original vaccine from 1921 was lost: BCG-Moreau (“Brazilian strain”: 1924), BCG-Russia (BCG-Moscow or “Russian strain”: 1924; genetically identical to BCG-Bulgaria or BCG-Sophia: 1950s), BCG-Japan (“Tokyo strain 172”: 1925), BCG-Romania (1925), BCG-Sweden (“Goethenburg strain”: 1926), BCG-Birkhaug (1927), BCG-Danish (BCG-Denmark or BCG-Copenhagen or “Danish strain 1331”: 1931), BCG-Tice (BCG-Chicago or “Tice strain”: 1934), BCG-Frappier (BCG-Montreal: 1937), BCG-Phipps (BCG-New York, BCG-Park, BCG-Philadelphia: 1938), BCG-Prague (“Czechoslovakian” strain: 1947), BCG-China (BCG-Beijing: 1947 or 1948), BCG-Shanghai (1948), BCG-Lanzhou (1948), BCG-Connaught (BCG-Toronto or “Theracys strain”: 1948), BCG-Polish (1950s), BCG-Glaxo (“BCG-London F10” or “Glaxo strain 1077”: 1954), BCG-Pasteur (“Pasteur strain 1173P2”: obtained in 1961), BCG-Mexico (1970), BCG-Mérieux (1989).

The following eight strains are the most common BCG strains in present use: Moreau, Russia, Japan, Danish, Tice, Connaught, Glaxo, and Pasteur. These five BCG strains represent more than 90% of the global BCG production: Russia, Japan, Danish, Glaxo, and Pasteur [16], [18]. According to Ritz et al., for some BCG strains (Russia, Japan, Danish, Prague, Glaxo, and Pasteur) results from at least nine studies were published from each strain, whereas for others, very little or no study results were found in the literature [15]. Studies and observations have shown that BCG-Pasteur and BCG-Danish are “strong” vaccines with higher immunogenicity and with greater complication rates than BCG-Japan or “weak” vaccines as BCG-Russia or BCG-Glaxo [18], [19].

Each of these BCG vaccines is produced in a different manner, and they are recognized to differ in various qualities, such as the proportion of viable cells per dose [5], [10]. However, the majority of the world's population is supplied with BCG vaccines procured by UNICEF (The United Nations Children's Fund) on behalf of the GAVI Alliance (formerly “Global Alliance for Vaccines and Immunization”). UNICEF uses only four BCG vaccine suppliers, who produce only three different BCG vaccine strains: BCG-Russia, BCG-Japan, and BCG-Danish [5].

BCG is also recognized to cause cross-protection against leprosy, as shown in a review [20] and in a meta-analysis [21]. That meta-analysis found that experimental studies demonstrated an overall protective effect of 26% (95% CI 14–37%) and that observational studies overestimated the protective effect [21]. Over the years, several vaccine trials using BCG have been performed to establish its limited protective effect against leprosy, often in combination with *M. leprae* or related mycobacterium vaccines. BCG was as good as, or superior to the other mycobacterium vaccines [22], [23].

Additionally, cross-protection of BCG against BUD was also shown in several studies, but their results are partially contradictory. An earlier clinical trial in Uganda showed an immune protection by BCG vaccination lasting six months [24]. The findings are consistent with another clinical trial in Uganda concluding that BCG vaccination provides only short-term protection against BUD [25]. In two studies in Benin, BCG was shown to be protective against more severe BUD, notably osteomyelitis [26], [27]. A study performed in Cameroon concluded that BCG appeared to protect children against more severe forms of BUD with multiple lesions [28]. However, none of these studies described the BCG strain used for vaccination.

In a mouse model experiment, the potential mechanisms for cross-protection were studied. A study identified and characterized the *M. ulcerans* homologue of the important protective mycobacterial antigen 85 (Ag85A) from BCG. This antigen was sufficiently conserved to allow cross-reactive protection, as demonstrated by the ability of *M. ulcerans*-infected mice to exhibit strong cellular immune responses to both BCG and its purified Ag85 complex [29]. It was also shown, that the BCG vaccine offered short-term protection against experimental footpad infections of mice with *M. ulcerans*, and that duration of this protection could not be prolonged by a booster vaccination [30]. Another experiment using a mouse model observed that BCG vaccination significantly delayed the onset of *M. ulcerans* growth and footpad swelling through the induction of an earlier and sustained IFN-γ triggered T cell response in the draining lymph node. BCG vaccination also resulted in cell-mediated immunity in *M. ulcerans*-infected footpads [31].

Two epidemiological studies, performed in Benin, could not find any evidence of a protective effect of routine BCG vaccination against BUD. In the second study, in persons aged >5 years, a BCG scar even resulted in a risk factor of 2.5 for BUD compared with those without a BCG scar [14], [32]. The first two epidemiological studies on the effectiveness of BCG vaccines on BUD performed in Ghana did not show any significant difference between cases and controls regarding their BCG vaccination status [33], [34]. None of these studies described the BCG strain used for vaccination.

Although many studies on the BCG vaccine were performed, the results regarding the vaccine's effectiveness against mycobacterial diseases including BUD differ immensely. Based on this unclear situation, the present case-control study was conducted with a large study population in the Democratic Republic of the Congo (DR Congo), Ghana, and Togo. In these three countries, only three different BCG strains were used since BCG was introduced from 1978 through 1984: BCG-Russia, BCG-Japan, and BCG-Danish. In the context of the EC-funded research project “BuruliVac” (FP7/2010–2013; grant agreement N° 241500), the aim of the present study is to evaluate possible protective effectiveness of routine BCG vaccination containing live attenuated bovine tuberculosis bacillus *M. bovis* on BUD in the DR Congo, Ghana, and Togo.

## Materials and Methods

### BuruliVac

BuruliVac was founded in 2009 as consortium of 16 European and African partners. As there is currently no existing vaccine lead candidate available, BuruliVac aimed to identify and develop new vaccine candidates of three different types: (1) Mycolactone-directed vaccines, (2) attenduated live vaccines, and (3) subunit protein vaccines. Furthermore, BuruliVac evaluated the resulting vaccine candidates using bioinformatics, applied genomics and proteomics, and subjected them to consecutive test systems. BuruliVac was funded by the European Commission under the 7th Framework Programme of the European Union [35].

### BCG in study countries

The present study was performed in the DR Congo, Ghana, and Togo. These three countries follow the WHO recommendations for routine immunization, which are part of their national immunization programs. This includes the advice to administer the one-time BCG vaccine intracutaneously, as soon as possible, either at birth or directly after, but not later than twelve months after birth, because at that age the vaccination is usually of limited benefit, although it is not harmful or contraindicated. Booster shots are not recommended [36]. The WHO estimates the BCG coverage rates in these three African countries as follows: 78% in the DR Congo, 98% in Ghana, and 97% in Togo [37].

### Study sites

This study consists of data collected at the following three sites, which are members of BuruliVac. The Institut Médical Evangélique (IME) de Kimpese in the DR Congo has implemented the “Project Ulcère de Buruli”. Since 1999, the General Reference Hospital (GRH) of the IME, located in the Songololo Territory, 220 km southwest of Kinshasa, regularly admits BUD cases. In 2004, the GRH launched a specialized BUD program offering in-patient treatment free-of-charge and supplementary aid. The principal aims of this project are the improvement of patient care for BUD patients admitted to the IME and the promotion of early community-based detection of suspected BUD cases. Patients and controls were recruited from Kimpese and Nsona-Mpangu health zones, both located in the Songololo Territory, Province of Bas-Congo [38], [39].

The Department of Medicine and the Kumasi Centre for Collaborative Research (KCCR) of the School of Medical Sciences at the Kwame Nkrumah University of Science and Technology (KNUST) are based in Kumasi, Ghana. They are involved with BUD in the areas of training, diagnostic confirmation, provision of specialist care for BUD patients in disease endemic districts, recruitment of patients and controls from the Ahafo Ano North, Asante Akim North, Atwima-Nwabiagya, and the Upper Denkvira districts, which are all within 70 km of the Ashanti regional capital Kumasi [40], [41].

The Centre Hospitalier Régional Maritime (CHR Maritime) in Tsévié, Togo, collaborates since 2007 with the German Leprosy and Tuberculosis Relief Organization, Togo office (DAHWT). This collaboration is supported by the Togolese National Buruli Ulcer Control Program (“Programme National de Lutte contre L'Ulcère de Buruli – Lèpre et Pian” [PNLUB-LP]), in the area of training, active case finding, laboratory confirmation, and treatment of BUD. In 2007, the CHR Maritime was appointed National Reference Centre for BUD in Togo [42], [43].

### BCG strains

In the DR Congo, BCG vaccination was routinely introduced in 1984. The following BCG strains were used for vaccinations: 1984–2003: BCG-Russia (equivalent to “BCG-Bulgaria”; produced by Bulbio [BB-NCIPD], Sofia, Bulgaria, and by Serum Institute of India); 2004: BCG-Japan (produced by Japan BCG Laboratory); 2005–2009: BCG-Japan (produced by Japan BCG Laboratory) and BCG-Russia (produced by Serum Institute of India); 2010–2011: BCG-Japan (produced by Japan BCG Laboratory); 2012: BCG-Japan (produced by Japan BCG Laboratory) and BCG-Russia (produced by Serum Institute of India); January to July 2013: BCG-Russia (equivalent to “BCG-Bulgaria”; produced by Bulbio [BB-NCIPD], Sofia, Bulgaria); August and September 2013: BCG-Russia (produced by Serum Institute of India); October 2013 to date: BCG-Bulgaria which is BCG-Russia (produced by Bulbio [BB-NCIPD], Sofia, Bulgaria).

In Ghana, BCG vaccination was routinely introduced in 1978. The following BCG strains were used for vaccinations: 2007: BCG-Danish (produced by Danish Statens Serum Institute); 2008–2009: BCG-Bulgaria = BCG-Russia (produced by Bulbio [BB-NCIPD]; 2010 to date: BCG-Japan (produced by Japan BCG Laboratory, Tokyo, Japan). Exact data on BCG strains used in Ghana from 1978 through 2006 are not available.

In Togo, BCG vaccination was routinely introduced in 1980. The following BCG strains were used for vaccinations: 2004: BCG-Japan (produced by Japan BCG Laboratory, Tokyo, Japan); 2004–2009: BCG-Russia (produced by Serum Institute of India); 2010 to date: BCG-Russia (equivalent to “BCG-Bulgaria”; produced by Bulbio [BB-NCIPD], Sofia, Bulgaria, and by Serum Institute of India). Exact data on BCG strains used in Togo from 1980 through 2003 are not available.

### Study design and definitions

In these three study sites, the recruitment of both BUD cases (among patients presenting with “clinically suspected” BUD lesions) and healthy controls was conducted. The present retrospective case-control study defined cases (CA) as patients affected by BUD, whose diagnosis was confirmed in laboratory by microscopy, IS *2404* polymerase chain reaction (PCR), or culture. Any CA had at least one positive test result. Patients who were “clinically suspected” (CS) for BUD, but without laboratory confirmation (i.e. none of the tests results was positive) were not considered in the study population. The controls (CO) were defined as healthy persons without any history of BUD in the past, who were in close relationship with the CA (see in next chapter).

### Study population

In the time period from February 2010 through April 2013, data from 1,335 individuals were collected. Out of them, 406 (30.41%) were CA, 103 (7.72%) were CS, and 826 (61.87%) were CO. From these data, 622 participants (128 CA: 20.58%; no CS; 494 CO: 79.42%) were from the DR Congo, 504 participants (196 CA: 38.89%; 65 CS: 12.90%; 243 CO: 48.21%) were from Ghana, and 209 participants (82 CA: 39.23%; 38 CS: 18.18%; 89 CO: 42.58%) were from Togo. Four CA from Ghana and one CA from Togo had unknown BCG status and were excluded out of the study.

Consequently, the study population was comprised of 1,227 participants (401 CA: 32.68%; 826 CO: 67.32%), including 622 from the DR Congo (128 CA: 20.58%; 494 CO: 79.42%), 435 from Ghana (192 CA: 44.14%; 243 CO: 55.86%), and 170 from Togo (81 CA: 47.65%; 89 CO: 52.35%). The 826 CO were in the following relationship with the CA: 225 (27.24%) were family members, 518 (62.71%) neighbors, 32 (3.87%) friends or classmates, and 51 (6.17%) were others or those with unspecified relationship.

### Data collection

Data collection was conducted by means of the WHO “BU01” form, and standardized project-specific “BuruliVac” laboratory data entry forms (Form S1). All socio-demographic, clinical, and laboratory data were entered in a web-based database specifically designed for the “BuruliVac” project [43]. Following WHO guidance, the categories of BUD were defined as follows: Category I were single lesions <5 cm in diameter; Category II were single lesions between 5 and 15 cm in diameter; Category III were single lesions >15 cm in diameter, multiple lesions, lesions at critical sites or osteomyelitis [44].

The BCG vaccination status was assessed from all CA and CO of the study population by examining both sides of the arms or shoulders, and if they presented a scar typical for vaccination with BCG or not, but not by documents such as vaccination certificates or hospital registers. Former studies that evaluated the presence or absence of BCG scars to determine vaccination status reported that scars develop in most vaccinated persons, with scarring rates of >80% [14], [45]–[47].

### Sample collection

In the DR Congo, fine needle aspirates were only collected from non-ulcerative lesions. Routinely, a direct smear was conducted at peripheral health centers from the first fine needle aspiration (FNA) and then the sample was stored in transport media (7H9 and PANTA liquid) and forwarded to IME for microscopy and culture. The second FNA (if possible) or a suspension was forwarded to the Institut National de Recherche Biomédicale (INRB) in Kinshasa via IME, where microscopy and IS *2404* real-time PCR was performed. Similar procedures were applied for swabs and tissue biopsies, however stored in semi-liquid transport medium (Dubos and PANTA semi-liquid).

In Ghana and Togo, diagnostic samples were collected according to standardized procedures [43]. Briefly, swabs were collected by circling the entire undermined edges of ulcerative lesions. Fine needle aspirates were collected from the center of non-ulcerative lesions or from undermined edges of advanced ulcerative lesions with scarred edges. Punch biopsy samples were only collected from advanced ulcers with scarred edges if fine needle aspirates were tested negative by PCR according to recent WHO recommendation [48].

Standardized specimen collection bags including swabs, biopsy punches, syringes and needles, slides, containers with transport media (700 µl [swab and punch biopsy samples], 300 µl [FNA samples] CLS [cell lysis solution, Qiagen, Hilden, Germany] for PCR samples; 4 ml PANTA transport medium for mycobacterial cultures [Ghana only]) and data entry forms were provided to the study sites in Ghana and Togo [49]–[57].

Samples for PCR analysis in CLS and for mycobacterial culture in PANTA transport medium were transported at ambient temperature in an upright position in custom-made specimen collection bags from the field to the laboratories from the two study sites in Ghana and one study site in Togo, within a maximum of 48 hours and stored at 4–8°C until further processing. Slides for microscopy were transported in slide boxes at ambient temperature to the laboratory.

### Laboratory diagnostics

Direct smears for microscopy were prepared from swab and fine needle aspirates at the laboratory (Ghana: KCCR; Togo: CHR Maritime), and were subjected to Ziehl-Neelsen staining. Slides were analyzed according to the WHO recommended grading system [56], [58] including quality assurance measures (re-reading of slides at INH and DITM). For PCR analysis, DNA was prepared using the Gentra Puregene DNA extraction kit (Qiagen) with minor modifications of the manufacturer's protocol [59], [60].

In the study site in the DR Congo, the Maxwell 16 DNA extraction procedure was carried out with the Maxwell 16 Tissue DNA Purification Kit and the Maxwell 16 Instrument, according to manufacturer's instructions: 200 µl of specimen was added to 200 µl of lysis buffer (10 mM Tris-HCl pH 7.5, 10 mM NaCl, 10 mM EDTA, 50 ml 10% SDS solution) and 10 µl proteinase K (20 mg/ml) and incubated overnight at 60°C in a shaker incubator. IS*2404* qPCR was performed on an Applied Biosystems 7500 Fast Real-Time PCR System using the method previously described by Fyfe et al. [61].

In the study sites in Ghana and Togo, the dry-reagent-based (DRB) IS *2404* PCR (INH, KCCR) was applied, accompanied by external quality assurance through IS *2404* qPCR at DITM. Briefly, for DRB-PCR the oligonucleotides MU5 and MU6 were lyophilized in reaction tubes. Illustra PuReTaq Ready-To-Go PCR beads (GE Healthcare, Munich, Germany) were added and dissolved in water before adding template DNA [50], [51], [60]. IS*2404* qPCR was performed as recently described using a BioRad CFX96 real-time PCR detection system [61], [62]. All PCR assays included negative extraction controls, as well as positive, negative (no template) and inhibition controls.

### Ethics statements

In Kimpese, the DR Congo, the ethical clearance was obtained through the “Comite d'Éthique” of the “Ecole de Santé Publique” of the University of Kinshasa (Ref. No. ESP/CE/057/2010). In Kumasi, Ghana, the ethical clearance was obtained through the Committee on Human Research Publication and Ethics of the College of Health Sciences of the Kwame Nkrumah University of Science and Technology (Ref. No. CHRPE/91/10). In Tsévié, Togo, the ethical clearance was obtained through the national Togolese ethics committee (“Comité de Bioéthique pour la Recherche en Santé”) at the University of Lomé (14/2010/CBRS) and the study was approved by the “Ministère de la Santé de la République Togolaise” Lomé, Togo (Ref. No. 0009/2011/MS/DGS/DPLET). All samples analyzed in this study were collected for diagnostic purposes within the EC funded research project “BuruliVac”. Written informed consent was obtained from all study participants, or their guardians if aged <18 years, according to the recommendations of the respective ethical committees. In case of illiterates, informed consents were countersigned by means of thumb prints.

### Statistical analysis

All data assessed at these three study sites were entered into the web-based database of BuruliVac and descriptively analyzed with Excel 2007 (Microsoft, Redmond, WA). The hypothesis of the present study was to evaluate associations between the presence of BCG scars (independent variable), which are caused by BCG vaccinations, and risk for BUD (dependent variable). Bivariate approximative tests (χ^2^-tests) and exact test (Fisher's tests) were conducted using EpiInfo, version 3.3.2. (Centers for Disease Control and Prevention, Atlanta, GA) and multiple logistic regression by Stata software, version 9.0. (Stata Corporation, College Station, TX) and. Significant differences were defined as p-values below 0.05.

## Results

### Baseline data of cases and controls

Among the study population of 1,227 individuals (401 CA and 826 CO) males comprised 45.56% (559), which was not significantly (*p* = 0.57) different between CA (44.39%: 178) and CO (46.13%: 381). Stratification by the three countries found no significant differences in the proportion of males among CA and CO. Among the 401 CA, the range of age was 1 to 78 years (y) and the median of age was 13 y (25% percentile: 8 y, 75% percentile: 27 y). Among the 826 CO, the range of age was 1 to 90 y and the median of age was 16 y (25% percentile: 9 y, 75% percentile: 30 y). Age distribution in CA and CO was significantly (*p* = 0.01) different, as the CA were younger than the CO: Age group (AG) 0–9 y (30.42% in CA vs. 26.63% in CO), AG 10–19 y (34.66% vs. 28.81%), AG 20–39 y (21.95% vs. 29.78%), and AG 40–90 y (12.95% vs. 14.77%). Stratified by the three countries, significant differences (*p*<0.01 each) of the proportions of these four AG among CA and CO were found in Ghana and Togo, but not in the DR Congo (*p* = 0.97) (Table 1).

**Table 1:** Baseline data of cases^a^ and controls^b^ from the Democratic Republic of the Congo (DR Congo), Ghana, and Togo, collected from February 2010 through April 2013.

Country	DR Congo			Ghana			Togo			Total		
BUD	CA (%)	CO (%)	Total (%)	CA (%)	CO (%)	Total (%)	CA (%)	CO (%)	Total (%)	CA (%)	CO (%)	Total (%)
	(%)	(%)	(%)	(%)	(%)	(%)	(%)	(%)	(%)	(%)	(%)	(%)
Total	128 (100)	494 (100)	622 (100)	192 (100)	243 (100)	435 (100)	81 (100)	89 (100)	170 (100)	401 (100)	826 (100)	1,227 (100)
	(10.43)	(40.26)	(50.69)	(15.65)	(19.80)	(35.45)	(6.60)	(7.25)	(13.85)	(32.68)	(67.32)	(100)
Male	55 (42.97)	211 (42.71)	266 (42.77)	85 (44.27)	124 (51.03)	209 (48.05)	38 (46.91)	46 (51.69)	84 (49.41)	178 (44.39)	381 (46.13)	559 (45.56)
Female	73 (57.03)	283 (57.29)	356 (57.23)	107 (55.73)	119 (48.97)	226 (51.95)	43 (53.09)	43 (48.31)	86 (50.59)	223 (55.61)	445 (53.87)	668 (54.44)
p value			0.96			0.16			0.54			0.57
AG^c^ 0–9 y	39 (30.47)	154 (31.17)	193 (31.03)	56 (29.17)	65 (26.75)	121 (27.82)	27 (33.33)	1 (1.12)	28 (16.47)	122 (30.42)	220 (26.63)	342 (27.87)
AG^c^ 10–19 y	41 (32.03)	149 (30.16)	190 (30.55)	66 (34.38)	87 (35.80)	153 (35.17)	32 (39.51)	2 (2.25)	34 (20.00)	139 (34.66)	238 (28.81)	377 (30.73)
AG^c^ 20–39 y	28 (21.88)	116 (23.48)	144 (23.15)	48 (25.00)	89 (36.63)	137 (31.49)	12 (14.81)	41 (46.07)	53 (31.18)	88 (21.95)	246 (29.78)	334 (27.22)
AG^c^ 40–90 y	20 (15.63)	75 (15.18)	95 (15.27)	22 (11.46)	2 (0.82)	24 (5.52)	10 (12.35)	45 (50.56)	55 (32.35)	52 (12.97)	122 (14.77)	174 (14.18)
p value			0.97			<0.01*			<0.01*			0.01*
No BCG^d^ scar	44 (34.38)	142 (28.74)	186 (29.90)	78 (40.63)	74 (30.45)	152 (34.94)	53 (65.43)	61 (68.54)	114 (67.06)	175 (43.64)	277 (33.54)	452 (36.84)
BCG^d^ scar	84 (65.63)	352 (71.26)	436 (70.10)	114 (59.38)	169 (69.55)	283 (65.06)	28 (34.57)	28 (31.46)	56 (32.94)	226 (56.36)	549 (66.46)	775 (63.16)
p value			0.22			0.03*			0.67			<0.01*
AG^c^ 0–9 y	39 (100)	154 (100)	193 (100)	56 (100)	65 (100)	121 (100)	27 (100)	1 (NA)	28 (100)	122 (100)	220 (100)	342 (100)
No BCG^d^ scar	15 (38.46)	42 (27.27)	57 (29.53)	7 (12.50)	6 (9.23)	13 (10.74)	13 (48.15)	0 (NA)	13 (46.43)	35 (28.69)	48 (21.82)	83 (24.27)
BCG^d^ scar	24 (61.54)	112 (72.73)	136 (70.47)	49 (87.50)	59 (90.77)	108 (89.26)	14 (51.85)	1 (NA)	15 (53.57)	87 (71.31)	172 (78.18)	259 (75.73)
p value			0.17			0.56			1.00^e^			0.16
AG^c^ 10–19 y	41 (100)	149 (100)	190 (100)	66 (100)	87 (100)	153 (100)	32 (100)	2 (NA)	34 (100)	139 (100)	238 (100)	377 (100)
No BCG^d^ scar	16 (39.02)	54 (36.24)	70 (36.84)	23 (34.85)	17 (19.54)	40 (26.14)	25 (78.13)	2 (NA)	27 (79.41)	64 (46.04)	73 (30.67)	137 (36.34)
BCG^d^ scar	25 (60.98)	95 (63.76)	120 (63.16)	43 (65.15)	70 (80.46)	113 (73.86)	7 (21.88)	0 (NA)	7 (20.59)	75 (53.96)	165 (69.33)	240 (63.66)
p value			0.74			0.03*			1.00^e^			<0.01*
AG^c^ 20–39 y	28 (100)	116 (100)	144 (100)	48 (100)	89 (100)	137 (100)	12 (100)	41 (100)	53 (100)	88 (100)	246 (100)	334 (100)
No BCG^d^ scar	7 (25.00)	31 (26.72)	38 (26.39)	32 (66.67)	51 (57.30)	83 (60.58)	5 (41.67)	26 (63.41)	31 (58.49)	44 (50.00)	108 (43.90)	152 (45.51)
BCG^d^ scar	21 (75.00)	85 (73.28)	106 (73.61)	16 (33.33)	38 (42.70)	54 (39.42)	7 (58.33)	15 (36.59)	22 (41.51)	44 (50.00)	138 (56.10)	182 (54.49)
p value			0.85			0.29			0.18			0.33
AG^c^ 40–90 y	20 (100)	75 (100)	95 (100)	22 (100)	2 (100)	24 (100)	10 (100)	45 (100)	55 (100)	52 (100)	122 (100)	174 (100)
No BCG^d^ scar	6 (30.00)	15 (20.00)	21 (22.11)	16 (72.73)	0 (NA)	16 (66.67)	10 (100)	33 (73.33)	43 (78.18)	32 (61.54)	48 (39.34)	80 (45.98)
BCG^d^ scar	14 (70.00)	60 (80.00)	74 (77.89)	6 (27.27)	2 (NA)	8 (33.33)	0 (0)	12 (26.67)	12 (21.82)	20 (38.46)	74 (60.66)	94 (54.02)
p value			0.34			0.10^e^			0.10^e^			<0.01*

NA Not applicable. No prevalence was shown if n <5.

a Cases were defined as patients affected by Buruli Ulcer Disease (BUD) whose diagnosis was laboratory confirmed by testing with microscopy, polymerase chain reaction (PCR), and culture.

Any case had a least one positive test result.

b Controls were defined as healthy persons having a close relationship with the CA.

c AG, age group in years (y).

d BCG, Bacillus Calmette-Guérin.

The only available vaccine against mycobacterial diseases, which contains live attenuated bovine tuberculosis bacillus (*Mycobacterium bovis*). In our study, the status after BCG vaccination was assessed from all cases and controls of the study population by controlling both sides of the shoulder, if they presented a typical “BCG scar”. Studies that evaluated the presence or absence of BCG scars to determine vaccination status reported that scars develop in most vaccinated persons.

e Fisher's exact test was used if at least one cell of contingency table was below 5.

### Lesions of cases

Among the 401 CA, 383 (95.50%) were detected with a single lesion, 15 (3.74%) with two lesions each, two (0.50%) with three lesions each, and one (0.25%) with four lesions. Out of them, 167 (41.65%) CA had non-ulcerative and 234 (58.35%) ulcerative lesions. The proportion of detected non-ulcerative lesions was as follows: nodules (74: 18.45%), plaques, (58: 14.46%), edema only (27: 6.73%), papules (7: 1.75%), and osteomyelitis (1: 0.25%).

Among the 401 CA, microscopy was performed for 399 (99.50%), PCR for 384 (95.76%), and culture for 159 (39.65%). The sensitivity of the three tests was as follows: PCR 97.14% (373/384), microscopy 69.42% (277/399), and culture 35.22% (56/159). Of 384 (95.76%) CA with known lesion sites, 2.86% (11/384) were on the face, 41.41% (159) on the upper limbs, 11.46% (44) on the trunk, and 44.27% (170) on lower limbs. The right lower limb (26.30%: 101) was significantly (*p*<0.01) more frequently affected than the left lower limb (17.97%: 69), whereas no significant differences where found between presence of lesions on the right and left side of the body for the face, upper limbs, or trunk.

### BCG scars of cases and controls

Among 401 CA, 175 (43.64%) had no BCG scar (CA_scar_), whilst 226 (56.36%) had BCG scar (CA_no_scar_). Among 826 CO, 277 (33.54%) had no BCG scar (CO_scar_), whilst 549 (66.46%) had BCG scar (CO_no_scar_). The proportion of those with a BCG scar was significantly (*p*<0.01) higher among the CO than among CA. When stratified by the three countries, a significant difference of the proportion of individuals with a BCG scar among CA and CO was only found in Ghana (*p* = 0.03), and not in the DR Congo (*p* = 0.22) or in Togo (*p* = 0.67) (Table 1).

Stratified by four age groups, a significantly higher proportion of those with a BCG scar among CO was only found in AG 10–19 y and AG 40–90 y (p<0.01 each). Stratified by the three countries and four age groups, a significantly higher proportion of those with BCG scar among CO was only found in Ghana in 10–19 y (p = 0.03) (Table 1). Multivariate analysis confirmed that the independent variables country (*p*<0.01), age (*p*<0.01), and status of BCG vaccination (*p* = 0.02) did significantly influence the dependent variable, if an individual develops BUD (CA) or not (CO).

Stratified by sex, a significantly higher proportion of those with a BCG scar among CO was only found among females (*p*<0.01), but not males (*p* = 0.09). When stratified by sex and by country, no significant difference of that proportion was found. After stratification by three countries, two sexes, and four age groups, no significant correlation was found between the presence of BCG scar and BUD status of individual (CA or CO).

### BCG scars and categories of cases

Among the 175 CA_scar_ representing 85.14% (149/175) and 226 CA_no_scar_, representing 77.43% (175/226) the BUD category was recorded. The proportions of CA with category I, II and III among CA_scar_ were respectively 48.99% (73), 41.61% (62), and 9.40% (14), whereas these proportions were respectively 60.57% (106/175), 27.43% (48), and 12.00% (21) among CA_no_scar_. Consequently, among the CA_scar_, the proportion of those with categories II and III was 51.01% (76), which was significantly (*p* = 0.04) higher than among CA_no_scar_ (39.43%: 69). Stratified by the three countries, no significant correlation was found between presence of BCG scar and categories (I or II/III). Among the CA_scar_, the proportion of those detected with multiple lesions was with 4.57% (8) not significantly (*p* = 0.94) higher than those of 4.42% (10) among the 226 CA_no_scar_ (Table 2).

**Table 2:** BCG^a^ vaccination and cases^b^ of BUD category^c^ I and category^c^ II/III from the Democratic Republic of the Congo (DR Congo), Ghana, and Togo, collected from February 2010 through April 2013.

Country	DR Congo			Ghana			Togo			Total		
Categories^c^	I (%)	II/III (%)	Total (%)	I (%)	II/III (%)	Total (%)	I (%)	II/III (%)	Total (%)	I (%)	II/III (%)	Total (%)
	(%)	(%)	(%)	(%)	(%)	(%)	(%)	(%)	(%)	(%)	(%)	(%)
Total	59 (100)	31 (100)	90 (100)	79 (100)	81 (100)	160 (100)	41 (100)	33 (100)	74 (100)	179 (100)	145 (100)	324 (100)
	(18.21)	(9.57)	(27.78)	(24.38)	(25.00)	(49.38)	(12.65)	(10.19)	(22.84)	(55.25)	(44.75)	(100)
No BCG^d^ scar	20 (33.90)	14 (45.16)	34 (37.78)	29 (36.71)	38 (46.91)	67 (41.88)	24 (58.54)	24 (72.73)	48 (64.86)	73 (40.78)	76 (52.41)	149 (45.99)
BCG^d^ scar	39 (66.10)	17 (54.84)	56 (62.22)	50 (63.29)	43 (53.09)	93 (58.13)	17 (41.46)	9 (27.27)	26 (35.14)	106 (59.22)	69 (47.59)	175 (54.01)
p value			0.30			0.19			0.21			0.04*
Male	21 (100)	17 (100)	38 (100)	36 (100)	35 (100)	71 (100)	18 (100)	18 (100)	36 (100)	75 (100)	70 (100)	145 (100)
No BCG^d^ scar	7 (33.33)	7 (41.18)	14 (36.84)	9 (25.00)	17 (48.57)	26 (36.62)	12 (66.67)	12 (66.67)	24 (66.67)	28 (37.33)	36 (51.43)	64 (44.14)
BCG^d^ scar	14 (66.67)	10 (58.82)	24 (63.16)	27 (75.00)	18 (51.43)	45 (63.38)	6 (33.33)	6 (33.33)	12 (33.33)	47 (62.67)	34 (48.57)	81 (55.86)
p value			0.62			0.04*			1.00			0.09
Female	38 (100)	14 (100)	52 (100)	43 (100)	46 (100)	89 (100)	23 (100)	15 (100)	38 (100)	104 (100)	75 (100)	179 (100)
No BCG^d^ scar	13 (34.21)	7 (50.00)	20 (38.46)	20 (46.51)	21 (45.65)	41 (46.07)	12 (52.17)	12 (80.00)	24 (63.16)	45 (43.27)	40 (53.33)	85 (47.49)
BCG^d^ scar	25 (65.79)	7 (50.00)	32 (61.54)	23 (53.49)	25 (54.35)	48 (53.93)	11 (47.83)	3 (20.00)	14 (36.84)	59 (56.73)	35 (46.67)	94 (52.51)
p value			0.30			0.94			0.09^e^			0.18
AG^c^ 0–9 y	15 (100)	11 (100)	26 (100)	23 (100)	24 (100)	47 (100)	18 (100)	6 (NA)	24 (100)	56 (100)	41 (100)	97 (100)
No BCG^d^ scar	7 (46.67)	6 (54.55)	13 (50.00)	2 (8.70)	5 (20.83)	7 (14.89)	8 (44.44)	3 (50.00)	11 (45.83)	17 (30.36)	14 (34.15)	31 (31.96)
BCG^d^ scar	8 (53.33)	5 (45.45)	13 (50.00)	21 (91.30)	19 (79.17)	40 (85.11)	10 (55.56)	3 (50.00)	13 (54.17)	39 (69.64)	27 (65.85)	66 (68.04)
p value			0.70			0.42^e^			1.00^e^			0.69
AG^c^ 10–19 y	19 (100)	11 (100)	30 (100)	23 (100)	29 (100)	52 (100)	17 (100)	12 (100)	29 (100)	59 (100)	52 (100)	111 (100)
No BCG^d^ scar	7 (36.84)	4 (36.36)	11 (36.67)	6 (26.09)	11 (37.93)	17 (32.69)	13 (76.47)	10 (83.33)	23 (79.31)	26 (44.07)	25 (48.08)	51 (45.95)
BCG^d^ scar	12 (63.16)	7 (63.64)	19 (63.33)	17 (73.91)	18 (62.07)	35 (67.31)	4 (23.53)	2 (16.67)	6 (20.69)	33 (55.93)	27 (51.92)	60 (54.05)
p value			1.00^e^			0.37			1.00^e^			0.67
AG^c^ 20–39 y	12 (100)	6 (100)	18 (100)	23 (100)	19 (100)	42 (100)	4 (NA)	7 (100)	11 (100)	39 (100)	32 (100)	71 (100)
No BCG^d^ scar	3 (25.00)	2 (33.33)	5 (27.78)	13 (56.52)	15 (78.95)	28 (66.67)	1 (NA)	3 (42.86)	4 (36.36)	17 (43.59)	20 (62.50)	37 (52.11)
BCG^d^ scar	9 (75.00)	4 (66.67)	13 (72.22)	10 (43.48)	4 (21.05)	14 (33.33)	3 (NA)	4 (57.14)	7 (63.64)	22 (56.41)	12 (37.50)	34 (47.89)
p value			1.00^e^			0.13^e^			1.00^e^			0.12
AG^c^ 40–90 y	13 (100)	3 (100)	16 (100)	10 (100)	9 (100)	19 (100)	2 (NA)	8 (100)	10 (100)	25 (100)	20 (100)	45 (100)
No BCG^d^ scar	3 (23.08)	2 (66.67)	5 (31.25)	8 (80.00)	7 (77.78)	15 (78.95)	2 (NA)	8 (100)	10 (100)	13 (52.00)	17 (85.00)	30 (66.67)
BCG^d^ scar	10 (76.92)	1 (33.33)	11 (68.75)	2 (20.00)	2 (22.22)	4 (21.05)	0 (NA)	0 (0)	0 (0)	12 (48.00)	3 (15.00)	15 (33.33)
p value			0.21^e^			0.91^e^			NA			**0.02** e *

NA Not applicable. No prevalence was shown if n <5.

a BCG, Bacillus Calmette-Guérin.

The only available vaccine against mycobacterial diseases, which contains live attenuated bovine tuberculosis bacillus (*Mycobacterium bovis*). In our study, the status after BCG vaccination was assessed from all cases and controls of the study population by controlling both sides of the shoulders, if they presented a typical “BCG scar” caused by intracutaneous BCG vaccination. Studies that evaluated the presence or absence of BCG scars to determine vaccination status reported that scars develop in most vaccinated persons.

b Cases were defined as patients affected by Buruli Ulcer Disease (BUD) whose diagnosis was laboratory confirmed by testing with microscopy, polymerase chain reaction (PCR), and culture.

Every case had a least one positive test result for BUD.

c Categories of BUD.

According to WHO, the categories of BUD were defined as follows: Category I correspond to single lesions with <5 cm in diameter; Category II correspond to single lesions between 5 and 15 cm in diameter; Category III correspond to single lesions with >15 cm in diameter, multiple lesions, or osteomyelitis.

d AG, age group in years (y).

e Fisher's exact test was used if at least one cell of contingency table was below 5.

Among individuals with known BUD category (149 CA_scar_ and 175 CA_no_scar_), the proportion of males was 44.75% (145), which was not significantly (*p* = 0.55) different between CA_scar_ (42.95%: 64) and CA_no_scar_ (46.29%: 81). Stratified by sex, no significant correlation was found between presence of BCG scar and categories (I or II/III). Among the CA_scar_, the range of age was 1 to 78 y and the median of age was 18 y. Among the CA_no_scar_, the range of age was 2 to 70 y and the median of age was 12 y. Age distribution in CA_scar_ and CA_no_scar_ was significantly (*p*<0.01) different, as the CA_scar_ were younger than the CA_no_scar_: AG 0–9 y (20.81% in CA_scar_ vs. 37.71% in CA_no_scar_), AG 10–19 y (34.23% vs. 34.29%), AG 20–39 y (24.83% vs. 19.43%), and AG 40–90 y (20.13% vs. 8.57%) (Table 2).

After stratification by the three countries, two sexes and four age groups, no significant correlation was found between presence of BCG scar and categories (I or II/III). Multivariate analysis confirmed, that the independent variables country (*p* = 0.31), sex (*p* = 0.24), age (*p* = 0.96), and presence of BCG scar (*p* = 0.07) did not significantly influence the dependent variable, if an individual develops BUD category I or category II/III.

### BCG scars and duration of BUD prior to first presentation

Among the 175 CA_scar_, the proportions of individuals with duration of 0–30 days (d), 31–60 d, 61–90 d, 91–180 d, and >180 d were 46.29% (81), 21.71% (38), 12.57% (22), 13.14% (23), and 6.29% (11), whereas these proportions were 46.02% (104), 22.12% (50), 13.72% (31), 11.06% (25), and 7.08% (16) among the 226 CA_no_scar_. The difference was not significant (*p* = 0.97), neither after stratification by the BUD categories.

### BCG scars and time to healing

Among the 401 CA, 305 (76.06%) were treated adequately by only antibiotics, 87 (21.70%) by antibiotics and surgery, seven (1.75%) by surgery only, and from two (0.50%) CA, no data on treatment were available. Among the 175 CA_scar_ representing 82.29% (144/175) the time to healing (i.e. the time difference between onset of treatment up to the point of time of macroscopic healing of BUD lesion) was known, by contrast with those of 80.97% (183/226) of 226 CA_no_scar_. Among the 144 CA_scar_, the proportions of time to healing of 7–90 d, 91–180 d, and >180 d were 27.08% (39), 45.83% (66), and 27.08% (39), whereas these proportions were 32.79% (60), 33.33% (61), and 33.88% (62) among the 226 CA_no_scar_. The difference was not significant (*p* = 0.07), and neither after stratification by the BUD categories of lesions.

## Discussion

This is one of the largest observational studies on the effectiveness of Bacillus Calmette-Guérin (BCG) vaccines on Buruli Ulcer Disease (BUD). The aim of the present retrospective case-control study was to evaluate possible protection of routine BCG vaccination with live attenuated bovine tuberculosis bacillus *Mycobacterium bovis* against BUD in the DR Congo, Ghana, and Togo. Since the first human vaccination with BCG in 1921, many studies of BCG vaccines have been performed to estimate their effectiveness, but their results differed immensely. These discrepancies are explained by three main factors: the BCG strain used for vaccination, the population vaccinated, and the mycobacterial disease or its manifestation.

The past and continued use of both strong and weak vaccine strains makes interpretation and comparison of clinical trials extremely difficult, thus no conclusions can be made that one BCG strain is clearly superior to another in the protection of humans against tuberculosis or other mycobacterial diseases [17], [63]. More than 20 different BCG seed strains are in use for vaccination, which are produced by more than 40 manufacturers. African countries like the DR Congo, Ghana and Togo, were mainly supplied with BCG vaccine procured by UNICEF as BCG-Russia, BCG-Japan, and BCG-Danish. As explained above, the BCG vaccines used in these three countries changed very often, so it was not possible to figure out retrospectively with which BCG strain a certain study participant was vaccinated if that person has shown a typical BCG scar. As no documentation in hospital files or on vaccination cards was performed, no data on exact time of vaccination could have been assessed. Consequently, the present study could not consider the BCG strain used for vaccination even though it is known that strong strains as BCG-Danish, less strong strains as BCG-Japan and weak strains as BCG-Russia were in use in these three countries. This classification refers only to tuberculosis and it is completely unknown if this might be also conferrable on BUD [17], [19].

This study assessed the effectiveness of BCG vaccination on BUD only. Tuberculosis, leprosy or any other disease which might influence the data, were not considered. The study population included 401 laboratory confirmed BUD cases and 826 adequate controls. To minimize confounding, the association between presence of BCG scar and BUD status (case or control) were calculated after stratification by the three countries, two sexes, and four age groups, and by multiple analysis.

Several studies have shown that the effectiveness of BCG is dependent on the population in which the vaccination is used. Age plays a role, as effectiveness among children is much higher in preventing tuberculous meningitis and miliary tuberculosis [12]–[14]. On the other hand, BCG vaccines seem to be more effective against leprosy among adults [20], [21]. To avoid influence of age, all analyses were performed after stratification by four age groups. The age distribution of cases in the present study was comparable with those in others [43], [53].

It is completely unknown if there is any age-depending vaccine effectiveness against BUD like found against tuberculosis and leprosy. After stratification into three countries and four age groups, the present study found only a significant higher proportion of those with BCG scar among CO in Ghana in AG 10–19 y (p = 0.03), but confounded by sex. After stratification by three countries, two sexes and four age groups, no significant correlation was found between the presence of BCG scar and BUD status of individual (CA or CO).

Furthermore, that vaccine effectiveness was calculated to be different in populations with high or low exposure to environmental mycobacteria. High exposure to mycobacteria affects the immune system in various ways and thus, BCG might not improve greatly upon that background [5], [8]. In the three study sites of the present study, it was assumed that there was equal, or at least comparable, exposure to mycobacteria among the populations. To avoid influence of country specific populations in general, all analyses were performed after stratification by the three countries.

In the present study, multivariate analysis has shown that country, sex, age, and presence of BCG scar did not significantly influence whether an individual develops BUD category I or category II/III. Furthermore, the status of BCG vaccination was also not significantly related to duration of BUD before initial presentation of patients nor to time of healing. These results underline those of four studies performed in Benin [14], [32] and in Ghana [33], [34], which did not reveal any significant difference between cases and controls regarding their BCG vaccination status. These results contradict those of two other studies performed in Benin which generated the hypothesis that BCG vaccination might protect children against more severe forms of BUD, notably osteomyelitis [26], [27], and another study performed in Cameroon which concluded that BCG appeared to protect children against more severe forms of BUD with multiple lesions [28]. None of the studies considered the BCG strain used for vaccination, and they could not answer the question if certain BCG strains might protect better than others against BUD.

The present study has the same limitation. Exact data on BCG vaccination among the study participants could not be assessed by documents, such as vaccination certificates or hospital registers. Thus, the status of BCG vaccination of every case and control was assumed by detection of a typical scar on one shoulder or anterior side of the forearm, based on the fact that scars develop in most vaccinated persons as described before [14], [45]–[47]. Probably a certain proportion of individuals were defined as “vaccinated”, even though the scar was caused by something other than a BCG vaccination (“false positive”). On the other hand, also a certain proportion might have been defined as “not vaccinated”, if no scar was found on the shoulder or anterior side of the forearm, because BCG vaccination did not lead to a “typical scar” (“false negative”). The number of such “false positive” and “false negative” cases and controls is not known and could not be estimated in the present study. Furthermore, no other data on the BCG vaccination (e.g. method of application, booster vaccination, and side effects) could be assessed. This inaccuracy cannot be estimated either, but might be equally distributed among cases and controls. To minimize this bias, we have chosen a case-control-design.

From the time since the first studies were conducted on the effectiveness of the BCG vaccine, the results are varying and will continue to vary as long as retrospective studies with little precise data are performed. As a consequence of this, we recommend to conduct prospective studies, with an exact documentation as to which vaccine was administered. Given the fact that some BCG strains might have a short-time protection against BUD in certain populations as shown in some studies [24], [25], this effect would have little impact on the overall incidence of BUD. A safe and effective specific vaccine with long-term protection against BUD which could be used in several populations of the most BUD endemic countries would be an adequate preventive tool to reduce the risk for this disease.

Given the fact that some BCG strains might provide protection to avoid more severe forms of BUD, notably osteomyelitis [26], [27] and multiple lesions [28], this effect would also not decrease the incidence of BUD, because only a small proportion of BUD cases are diagnosed with osteomyelitis (in the present study <1%) and only a small proportion of BUD cases are diagnosed with multiple lesions (in the present study <5%).

Even though only a limited number of studies on BCG effectiveness for the prevention of BUD have been conducted, the probability of finding an effective BCG strain against BUD is low, and thus efforts to research specific vaccines against BUD should be accelerated like approached by the BuruliVac consortium.

In our study, we did not observe significant evidence of a protective effect of routine BCG vaccination with *Mycobacterium bovis* on the risk of developing either BUD or severe forms of BUD. Since accurate data on BCG strains are used in these three countries were not available, no final conclusion can be drawn on the effectiveness of BCG strain in protecting against BUD. As has been suggested for tuberculosis and leprosy, well-designed prospective studies on different existing BCG vaccine strains are needed also for BUD and further research on safe and specific vaccines against BUD should be supported.

## Supporting Information

S1 Checklist **STROBE checklist for case control studies.**
(DOC)
