# Supplementary material for: Effectiveness of Routine BCG Vaccination on Buruli Ulcer Disease: A Case-Control Study in the Democratic Republic of Congo, Ghana and Togo
Source: PLoS Negl Trop Dis. 2015 Jan 8;9(1):e3457. doi: 10.1371/journal.pntd.0003457 (PMC4287572; doi:10.1371/journal.pntd.0003457)
Supplement: S1 Checklist — STROBE checklist for case control studies. (DOC) [file pntd.0003457.s001.doc]

STROBE Statement—Checklist of items that should be included in reports of ***case-control studies***

|  | Item No | Recommendation |
| --- | --- | --- |
| **Title and abstract** | 1 | (*a*) Done. See title (page 1). |
|  |  | (*b*) Done. See abstract (page 2). |
| Introduction | | |
| Background/rationale | 2 | Done. See introduction (page 4-7). |
| Objectives | 3 | Done. See last paragraph of the introduction (page 7). |
| Methods | | |
| Study design | 4 | Done. See materials and methods (page 7-13). |
| Setting | 5 | Done. See materials and methods (page 7-13). |
| Participants | 6 | Done. See study population (page 10). |
| Variables | 7 | Done. See materials and methods (page 7-13). |
| Data sources/ measurement | 8* | Done. See materials and methods (page 7-13). |
| Bias | 9 | Done. See materials and methods (page 7-13), results (page 13-17), discussion (page 17-20). |
| Study size | 10 | Done. See materials and methods (page 7-11). |
| Quantitative variables | 11 | Done. See materials and methods (page 7-11). |
| Statistical methods | 12 | Done. See materials and methods (page 7-11), especially statistical analysis (page13). |
| Results | | |
| Participants | 13* | Done. See results (page 13-17). |
| Descriptive data | 14* | Done. See results (page 13-17). |
| Outcome data | 15* | Done. See results (page 13-17). |
| Main results | 16 | Done. See results (page 13-17). |
| Other analyses | 17 | Done. See results (page 13-17). |
| Discussion | | |
| Key results | 18 | Done. See discussion (page 17-20). |
| Limitations | 19 | Done. See discussion (page 17-20). |
| Interpretation | 20 | Done. See discussion (page 17-20). |
| Generalisability | 21 | Done. See discussion (page 17-20). |
| Other information | | |
| Funding | 22 | The authors appreciate the financial support for BuruliVac from the fund of the European Commission under the 7^th^ Framework Programme of the European Union. There is no conflict of interest. |

*Give information separately for cases and controls.

**Note:** An Explanation and Elaboration article discusses each checklist item and gives methodological background and published examples of transparent reporting. The STROBE checklist is best used in conjunction with this article (freely available on the Web sites of PLoS Medicine at http://www.plosmedicine.org/, Annals of Internal Medicine at http://www.annals.org/, and Epidemiology at http://www.epidem.com/). Information on the STROBE Initiative is available at http://www.strobe-statement.org.
